# Evidence for the Late MMN as a Neurophysiological Endophenotype for Dyslexia

**DOI:** 10.1371/journal.pone.0034909

**Published:** 2012-05-14

**Authors:** Nina Neuhoff, Jennifer Bruder, Jürgen Bartling, Andreas Warnke, Helmut Remschmidt, Bertram Müller-Myhsok, Gerd Schulte-Körne

**Affiliations:** 1 Department of Child and Adolescent Psychiatry, Psychosomatics and Psychotherapy, University of Munich, Munich, Germany; 2 Department of Child and Adolescent Psychiatry and Psychotherapy, University of Würzburg, Würzburg, Germany; 3 Department of Child and Adolescent Psychiatry and Psychotherapy, University Hospital Gießen and Marburg, Marburg, Germany; 4 Statistical Genetics Max Planck Institute of Psychiatry, Munich, Germany; University of Leicester, United Kingdom

## Abstract

Dyslexia affects 5–10% of school-aged children and is therefore one of the most common learning disorders. Research on auditory event related potentials (AERP), particularly the mismatch negativity (MMN) component, has revealed anomalies in individuals with dyslexia to speech stimuli. Furthermore, candidate genes for this disorder were found through molecular genetic studies. A current challenge for dyslexia research is to understand the interaction between molecular genetics and brain function, and to promote the identification of relevant endophenotypes for dyslexia. The present study examines MMN, a neurophysiological correlate of speech perception, and its potential as an endophenotype for dyslexia in three groups of children. The first group of children was clinically diagnosed with dyslexia, whereas the second group of children was comprised of their siblings who had average reading and spelling skills and were therefore “unaffected” despite having a genetic risk for dyslexia. The third group consisted of control children who were not related to the other groups and were also unaffected. In total, 225 children were included in the study. All children showed clear MMN activity to/da/−/ba/contrasts that could be separated into three distinct MMN components. Whilst the first two MMN components did not differentiate the groups, the late MMN component (300–700 ms) revealed significant group differences. The mean area of the late MMN was attenuated in both the dyslexic children and their unaffected siblings in comparison to the control children. This finding is indicative of analogous alterations of neurophysiological processes in children with dyslexia and those with a genetic risk for dyslexia, without a manifestation of the disorder. The present results therefore further suggest that the late MMN might be a potential endophenotype for dyslexia.

## Introduction

Dyslexia is a specific disorder in learning to read and spell despite normal intelligence, adequate schooling, and no obvious sensory deficits [1]. With 5%–10% of school-aged children affected, dyslexia is one of the most common learning disorders [2], [3].

Aside from reading and spelling deficits, a number of neurophysiological studies have revealed altered auditory event-related potentials (AERP) in both children and adults with dyslexia when passively discriminating between two phonemes, such as/da/and/ba/(for review see [4], [5], [6]). The AERP component which is related to this type of speech processing is the mismatch negativity (MMN). MMN is a pre-attentive measure of the AERP and reflects both the obligatory response to successful discrimination between two acoustic stimuli presented in succession and short-term auditory memory capacity. The MMN is a negative curve which is obtained by subtracting the AERP to a frequently presented standard stimulus from the AERP of an infrequently presented deviant stimulus. This negativity is registered at the fronto-central and central scalp electrodes, peaking around 150–250 ms from change onset [6], [7] and originates from sources in auditory and frontal cortices [6], [8]. The MMN is an objective measurement of the speech discrimination ability, and is particularly well-suited for studies in children; because active attention to the speech stimuli is not required [6].

A late MMN component (also referred to as the late discriminatory negativity or LDN [9], [10]) at a timeframe from 300–600 ms over fronto-central sites has also been described [10], [11], [12], [13], [14], [15], [16], [17], [18], [19]. It is mainly elicited by complex auditory stimuli like syllables and words, however it also occurs for tones [20]. Hommet et al. (2009) investigated sink and source patterns of the late MMN in children and located generators primarily in centro-parietal areas of the right hemisphere. The authors did not find an involvement of the supratemporal auditory cortex. Overall, the characteristics of the late MMN suggest the involvement of other brain processes than those attributed to the early MMN. It is thought to be associated to higher cognitive processes, such as attention related processes [21], letter-speech sound integration [13] and long term memory [22].

Most studies on MMN and dyslexia have focused on the early MMN, and deficits to speech sounds have been generally reported (for review see [4], [5]), although not always [18], [23], [24], [25], or only in subgroups [26], [27]. Bishop [5] states that studies on (early) MMN to speech sounds and dyslexia often suffer from low effect sizes, perhaps as a result from the employment of heterogeneous and small groups. Furthermore, the early MMN is often absent in a large percentage of healthy study participants and shows a very poor reliability on an individual level (ie. [28], [29]. Altogether, the efficacy of early MMN in the study of language disorders has not been fully established and many questions remain to be answered. None-the-less, the literature on dyslexia, early MMN and speech sound processing suggests that a large number of individuals with dyslexia will show reduced early MMN amplitude. Studies have also reported reduced late MMN amplitudes in dyslexic individuals [17], [18], [30]; [14], [18], [31], [32]; [12], [33]. Because the functional significance of the late MMN is less well understood it is not yet clear what factors might underlie late MMN deficits.

Finally, dyslexia is also a disorder with a complex and heterogeneous genetic basis [34], [35]. Four genes associated with dyslexia in particular are involved in the development of the cerebral neocortex, either in terms of axonal guidance (*ROBO1* [36]) or neuronal migration (*KIAA0319* [37], *DCDC2* [38], and *DYX1C1* [39]). Importantly, these genes are expressed in cortical brain regions that are part of the complex neuronal network for reading [40], [41]. Among these are the temporo-parietal cortices, the occipito-temporal cortices, and the inferior frontal cortex [42]. All of these brain areas have been found to be differentially activated in subjects with dyslexia. Specifically, in the left temporo-parietal region reduced activity correlated with phonological processing (e.g. rhyme detection and segmentation) and word reading [43], [44], [45]. Increased activation of the left inferior frontal area was associated with articulation in dyslexic subjects and was attributed to compensatory activation [43], [44]. Finally, abnormal activity reported in left-occipital temporal areas to word and pseudoword stimuli suggests a visual word processing deficit [41], [46], [47], [48], [49].

One major difficulty in dyslexia research is defining and characterizing dyslexia. This problem is inherently linked to the genetic heterogeneity of dyslexia [35] which contributes to the complex behavioural profiles observed. For example, many but not all dyslexic individuals present with speech processing deficits [5], phonological deficits or rapid naming deficits [50]. Further complicating the matter is that many, but not all, dyslexic individuals also show non-language related problems such as temporal processing deficits [51] or even arithmetic [52] and motor deficits [53]. These deficits or the lack of them seem to occur in no particular pattern, thus making it extremely difficult to acquire heterogeneous samples for investigation and indeed to describe and quantify dyslexia per se. Furthermore, the diagnosis of dyslexia is determined for study and clinical purposes based on behavioural criteria (i.e. low reading and/or spelling scores) in children. Adults are often classified retrospectively or according to non-standardized reading measures.

It is apparent that numerous genes will contribute in small ways to the manifestation of dyslexia, and the genetic profiles will differ from one group or individual to the next. Although candidate genes have been identified in dyslexia, and much has been understood about brain (dys)function in dyslexia it remains unclear how genetics impact brain function and how these areas are related to reading and spelling phenotypes.

The identification of endophenotypes has been proposed to bridge the gap between the genes involved in disorder pathophysiology and overt behavioural phenotypes (i.e. reading). Endophenotypes are intermediate phenotypes which are under strong genetic influence and present in the majority of individuals with a disorder. Because of their relevance for genetics, family members who are not affected by the disorder will show the endophenotype more frequently than control groups [44]. Endophenotypes can take on a number of forms, for example hormonal, anatomical or as investigated in the present study, electrophysiological. Endophenotypes more closely indicate “disorder” than overt phenotypes and are therefore substantially more straightforward to use for investigation purposes [54]. For example, using an endophenotype as inclusion criteria for a genetic study, as opposed to overt phenotypes, increases the likelihood of identifying genes related to the disorder. Understanding which genes are involved in a disorder has many consequences, including: implications for diagnostics; illumination of disorder heterogeneity; influencing the development of animal models to study disorder pathology; and the creation of early interventions for those individuals presenting with a particular endophenotype.

So far, endophenotypes have not been identified in dyslexia. However, two molecular genetic studies on dyslexia have investigated both early and late MMN elicited by speech stimuli as candidate endophenotypes for dyslexia. Both studies were able to detect a relationship of the late MMN to gene loci, but did not find any evidence for an influence of genetics on the early MMN [12], [33] suggesting that late MMN might be under genetic influence. Roeske et al. (2011) were able to show how the late MMN was significantly associated to *SLC2A3,* a gene on chromosome 12 which had not yet been associated with dyslexia. The functionality of *SLC2A3* renders it a compelling candidate for developmental disorders, as it is the predominant facilitative glucose transporter in neurons during child development. In a subsequent study [12] the late MMN was associated to rare variants between the prominent dyslexia candidate genes *KIAA0319* and *DCDC2,* both located on chromosome 6. Together, these findings suggest that the late MMN component in dyslexia is influenced by genetics. Thus, the neurophysiological correlates of speech perception in dyslexia which are under genetic influence might be mainly related to later cognitive processes. Taken together, there are a number of convincing reasons to further explore MMN components as possible endophenotypes for dyslexia.

### Present Study

We investigated both early and late MMN related to speech sound perception in dyslexic children and their unaffected siblings in order to further explore possible genetic influences on these components. To study these differences we employed the speech stimuli/ba/and/da/in a passive oddball paradigm. These same stimuli were used in earlier studies [17], [18], [23]. A small unrelated control group was included (see discussion for limitations).

Our primary goal was to determine if early and/or late MMN to speech sounds differed in dyslexic children and their unaffected siblings. Based on our previous findings [33], [55] we did not expect to find any evidence to suggest that early MMN is influenced by the genetics underlying dyslexia. We expected to find a reduced late MMN in both the dyslexic and the unaffected sibling groups compared to the control group.

## Methods

The children participating in this study were selected via a single proband sib-pair approach. The siblings were recruited from 2001–2004 at the Departments of Child and Adolescent Psychiatry and Psychotherapy at the Universities of Marburg and Würzburg in Germany, and was funded by DFG (Deutsche Forschungsgemeinschaft). Written informed consent was given by the parents for all participating children, and by the children themselves and the study was approved by the local ethic committees of the Universities of Marburg and Würzburg. All children were compensated with 10€ for taking part in the study. The families coming to the clinic were also refunded for transportation costs.

Children were selected for this study when at least two siblings were available and one sibling fulfilled the inclusion criterion of a discrepancy of ≥1 SD between the observed spelling score and that expected from the non-verbal IQ to be able to compare dyslexic children and there unaffected siblings [56]. Spelling was measured using an age-appropriate spelling-test (writing to dictation) [57], and an observed spelling score was calculated on the basis of a correlation of 0.4 between the proband’s IQ (measured by using the Culture Fair Test) and spelling [58]. Because there were no standardized German reading tests for children at or above the 5^th^ grade at the time of the study, a non-standardized reading test was performed with these children. This test requires children to read a list of 48 words as accurately and quickly as possible. The dependent variable was time needed to read words. All children included in the dyslexic group also fulfilled the criterion of a discrepancy of ≥1 SD between the observed reading performance and that expected from the non-verbal IQ. Although the probands with dyslexia in the current study had significantly poorer reading skills, they were recruited based on their below average spelling skills. Recruiting based on spelling disorder in German is often done because the German language represents a very transparent orthography. This transparency fosters reading skills, as phoneme-grapheme correspondences are very consistent [59]. Therefore, it is quite typical in German dyslexic populations to observe normal reading accuracy with potential fluency (speed) deficits [60], [61], [62], [63]. Spelling on the other hand remains difficult in German and these deficits are more persistent in the dyslexic populations [63]. From the total sample of 390 probands [64], [65] and their siblings, only affected probands and their matched unaffected siblings were chosen for the analysis. If any proband fulfilled the diagnostic criteria of ADHD the family was excluded from the study since their inclusion could have introduced further heterogeneity into the analysis. Additional exclusion criteria were a bilingual education, a non-verbal IQ*<*85, an uncorrected disorder of peripheral hearing or vision, and a psychiatric or neurological disorder influencing the development of reading and spelling ability [66].

The control group was recruited from a public school based on both a comparable school grade and age, and based on the same inclusion and exclusion criteria as mentioned above. In total a group of 225 children, aged 10–15 years, could be included in this present study (Table 1).

**Table 1:** Sample description, presenting the mean and standard deviation for sample number, age, spelling, reading, IQ, and handedness.

Sample	Dyslexic children	Siblings	Controls
N	105 (♂ 64, ♀ 41)	105 (♂ 26, ♀ 79)	15 (♂ 4, ♀ 11)
Age (years)	11.54 (1.65)	12.37 (2.11)	12.53 (0.33)
Spelling (T scores)	31.41 (5.63)	49.15 (6.85)	52.87 (5.07)
Reading (time, sec)	38.42 (10.33)	51.36 (10.22)	56.53 (12.08)
IQ	109.62 (11.84)	109.82 (12.85)	106.33 (7.5)
Handedness	91 right, 14 left	94 right, 11 left	15 right

In order to measure MMN a passive oddball paradigm was used which presented the consonant-vowel stimuli/da/and/ba/binaurally via headphones. The stimuli were synthetic speech stimuli synthesized with the Computerized Speech Research Environment (Computerized Speech Research Environment (CSRE) (1995) London: AVAAZ Innovations, Inc). The standard stimulus was/da/(85%) and the deviant stimulus was/ba/(15%). For both stimuli, stimulus duration was 240 ms. Stimuli were presented in a pseudorandom order with at least five standards between two deviants with a stimulus-onset asynchrony (SOA) of 980 ms [18], [19], [32]. The children were instructed to ignore the presented stimuli and their attention was directed towards a silent movie. Thirty-two electrodes (Fp1, Fp2, F7, F3, Fz, F4, F8, FT7, FC3, FCz, FT8, T3, C3, Cz, C4, T4, TP7, CP3, CP4, TP8, T5, P3, Pz, P4, T6, O1, Oz, O2 and four EOG-electrodes) were placed on the scalp, based on the expanded international 10/20-system, with reference to the left mastoid. The EEG was re-referenced offline to averaged mastoids, the ground electrode was positioned at Fpz. Eye movements were detected with electrodes placed above, below and next to the subject’s eyes. The EEG was amplified with Neuroscan Amplifiers. EEG-recording was continuous and A/D converted at a sampling rate of 256 Hz. The recorded EEG was filtered with a 0.53–40 Hz band pass using Brainvision Analyzer. Eye artefacts were corrected by performing an independent component analysis (ICA), with manual identification and exclusion of the eye artefact components. Further artefacts were removed by excluding trials automatically with two gradients (allowed maximum of 50 µV per sample point; maximum allowed absolute difference 150 µV in 200 ms) and max-min criteria (maximum amplitude of +−100 µV). Signals were averaged into epochs of 1100 ms, including a pre-stimulus baseline of 100 ms.

**Figure 1:**
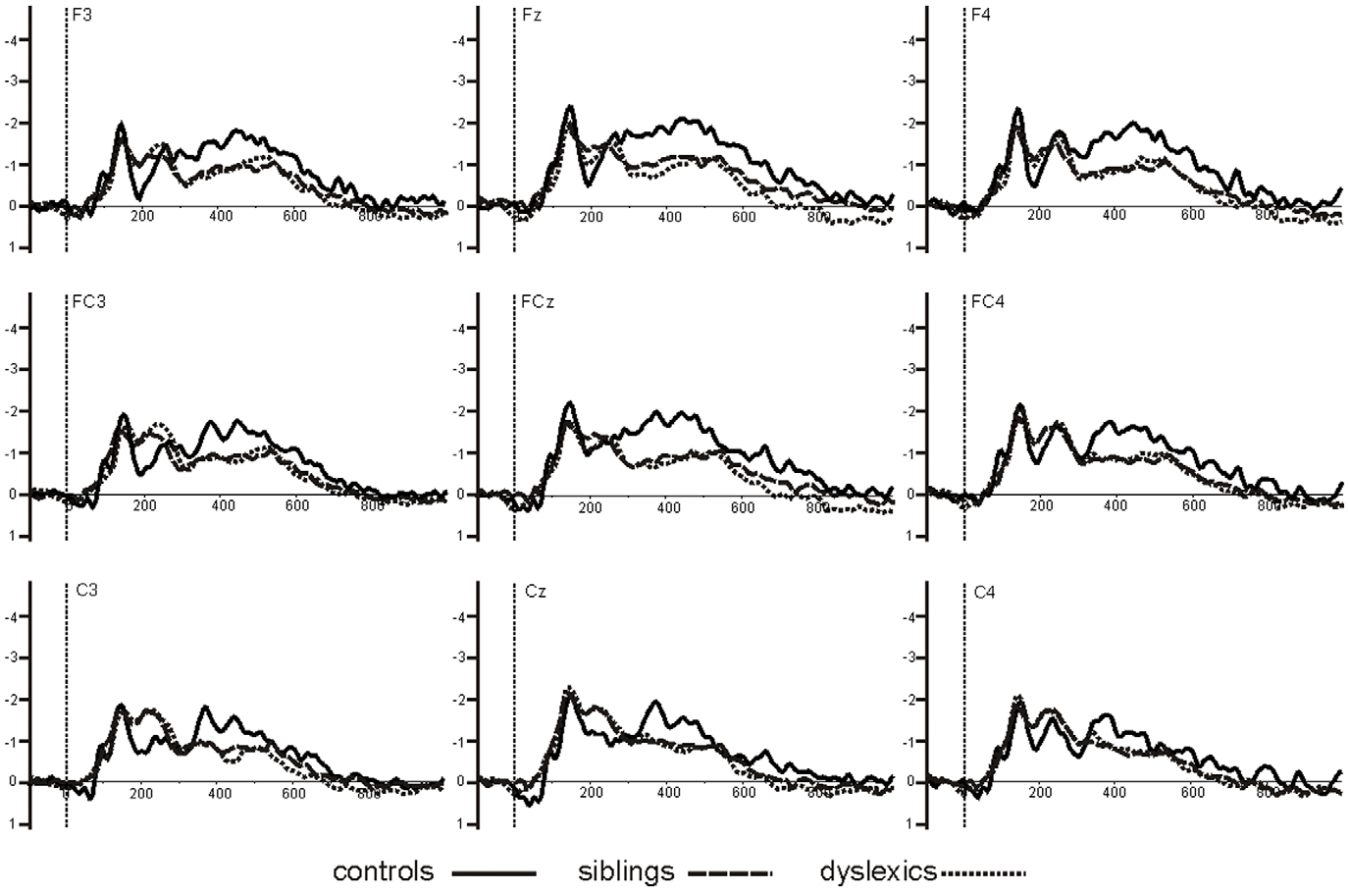
MMN1, MMN2, and late MMN for all 3 groups. MMN1, MMN2, and late MMN for dyslexic children (black line), unaffected siblings (dashed line), and unrelated controls (dotted line) at the nine fronto-central electrodes, giving the timeframes for the MMN1 (84–188 ms), MMN2 (188–300 ms), and late MMN (300–700 ms).

The average accepted trials was 271 for deviant stimuli and 372 for standard stimuli. The lowest number of accepted trials for any proband was 47. Therefore, all children had an acceptable number of accepted trials and we did not exclude any children from the analysis. Difference waveforms (MMN curves) were calculated by subtracting the averaged standard from the averaged deviant AERP. Grand averages were generated over all subjects for each group separately. Based on the observed scalp topography of the MMN in the control group and on electrode choice in previous MMN studies, the following fronto-central electrodes were chosen for analysis: F3, F4, Fz, C3, C4, Cz, Fc3, Fc4, Fcz. This fronto-central region is also known to be of interest for auditory stimulus perception and processing [67] and these electrodes were also used for group comparisons between dyslexic probands and controls in former speech perception studies [18], [19]. The grand average wave forms (figure 1) revealed three MMN components: labelled MMN1, MMN2, and late MMN. For the analysis of these components the grand averages were tested against zero using running t-tests in order to determine which time windows differed significantly from zero for each component. The following three time windows were determined: MMN1 (84–188 ms), MMN2 (188–300 ms) and late MMN (300–700 ms). Mean MMN peak amplitude and mean MMN peak latency for MMN1 and MMN2 were calculated using these time windows. Because late MMN revealed a broad amplitude with no obvious peak the value of the area under the curve (µV *ms) was taken.

We introduced a random factor variable into our statistical model to account for the dependency (familial relationship) between the probands with dyslexia and their unaffected siblings. Furthermore, due to the large differences in sample sizes we used a PQL method (penalized quasi likelihood) which is robust for small sample sizes. Age, IQ and sex were modelled as covariants. Independent sample t-tests were run over the groups “family” and “control” for mean peak amplitude and mean peak latency for MMN1 and MMN2 and for mean area for the late MMN.

## Results

MMN was generated for all three groups (dyslexic children, unaffected siblings, and unrelated controls) and revealed three distinct time windows (MMN1, MMN2, and late MMN) as can be seen in Fig. 1 and Table 2.

**Table 2:** Means and standard deviations of the MMN peak amplitudes, areas and latencies.

	DyslexicsMeans (SD)	SiblingsMeans (SD)	ControlsMeans (SD)
MMN1, amplitude	−2.93 µV (1.65)	−2.84 µV (1.39)	−2.64 µV (1.25)
MMN2, amplitude	−2.75 µV (1.50)	−2.661 µV (1.38)	−2.48 µV (1.10)
late MMN (area under curve)	−260 µV*ms(412)	−350 µV*ms (376)	−480 µV*ms (276)
MMN1, latency	144.68 ms (19.92)	142.40 ms (21.18)	134.70 ms (24.14)
MMN2, latency	236.72 ms (28.01)	233.94 ms (28.06)	251.50 ms (26.56)

### MMN1 and MMN2

Both MMN1 and MMN2 peaks were clearly visible in all three groups. We found no differences between groups for mean amplitude (MMN1: *p* = .63; MMN2: *p* = .82) or peak latency (MMN1: *p* = .99; MMN2: *p* = .88).

### Late MMN

**Figure 2:**
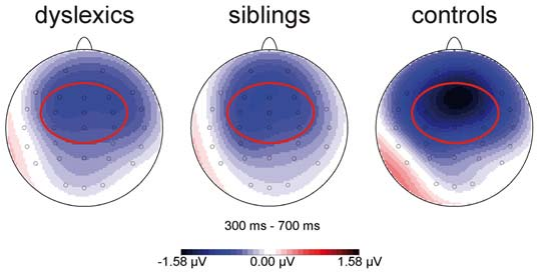
Topographic maps for the late MMN. Topographic maps for the time period 300–700 ms (late MMN) for dyslexic children, unaffected siblings, and unrelated controls, showing the ROI encircled in red.

Control children revealed significantly greater late MMN than dyslexic and unaffected siblings (*t*(117) = −2.38, *p*<.02, d = −.64. Figure 2 depicts the scalp topography of the late MMN. In all three groups the greatest activity can be seen over the fronto-central electrode sites, however the activity is greater in the control group.

## Discussion

This present study was conducted to obtain further insight on the significance of early and late MMN elicited by differences in speech sounds as potential neurophysiological endophenotypes for dyslexia. For this purpose, children with a diagnosis of dyslexia, their unaffected siblings and unrelated control children were compared using a passive oddball paradigm with consonant-vowel stimuli.

We found three distinct MMN components of which the late MMN was reduced both in children with dyslexia and their unaffected siblings in comparison to control children. The early MMN components did not differentiate the groups. The first MMN component emerged in an atypical time window for MMN. Some studies on speech perception in dyslexia have reported group differences at similar early latencies (at 130 ms) [68], whereas others have not [18], [69]. The second MMN component was typical of the early MMN latency. The amplitude of this component can be reduced in dyslexia, (for reviews see [4], [6], but this has not always been found [18], [28], [29], [70]. The present findings suggest that early MMN is not under genetic influence in dyslexia, which is supported by our previous research [33], [55].

### Late MMN: A Candidate Endophenotype for Dyslexia

Both the latency and scalp topography of the late MMN in the present study is consistent with reports in previous studies. These studies attributed the significance of the late MMN to higher cognitive processes, such as attention related processes [21], letter-speech sound integration [13] and to long term memory [22] as opposed to the detection of speech sound differences as associated to the MMN [6].

Previous studies have also found an attenuated late MMN to speech sounds in dyslexia [11], [12], [14], [17], [19], [33], [70]. However, this is the first report of attenuation of an ERP in relatives of dyslexic individuals, who therefore have a genetic risk for dyslexia, but have not developed reading and/or spelling disorders. The present findings complement recent molecular genetic research by our group [12], [33] and further suggest that the late MMN might be a viable endophenotype for dyslexia research.

The identification of genes that contribute to a susceptibility to complex neuropsychiatric disorders is generally not successful when conventional genetic approaches are employed. Using endophenotypes (e.g. as study inclusion criteria) to investigate disorders with a complex genetic basis should aid molecular genetic studies because endophenotypes are more directly under genetic influence than the complex behaviours used to classify and diagnose psychiatric disorders (such as dyslexia, depression, schizophrenia and dementia). So far, research on the late MMN and dyslexia suggest that the area of late MMN to speech sounds might fulfil three criteria for endophenotype classification as suggested by Gottesman & Gould (2003): 1) the late MMN amplitude has been shown to be associated with dyslexia [11], [14], [17], [18], [19], 2) it has been associated with the genetics of dyslexia (for both known (*DCDC2* and *KIAA0319*) [12] and novel (*SLC2A3*) [33] candidate genes), and 3) as the present study demonstrates, it is also attenuated in individuals with a genetic risk for dyslexia, but who did not develop reading and spelling deficits.

Taken together, these findings suggest that the late MMN is influenced by the underlying genetics of dyslexia. Finding reduced late MMN in siblings with and without dyslexia opens the field for further investigations that might address protective environmental factors or compensatory mechanisms. Furthermore, future investigations of late MMN and dyslexia might reveal new insight for dyslexia interventions, since unaffected siblings master a normal reading and writing level even with reduced neurophysiological answers to speech sounds.

Despite these promising results, whether the late MMN can be classified as an endophenotype for dyslexia and if it can be concretely employed for future research still needs to be systematically examined. Although the late MMN seems to be a promising candidate for an endophenotype in dyslexia, replication of the present findings, as well as our previous findings [12], [33] is essential and substantiation of the late MMN’s heritability, occurrence throughout dyslexic families and presence after compensation or remediation remains to be established. In general, the identification of endophenotypes and the subsequent understanding of the genetics contributing to dyslexia have the potential to pave the way for improving diagnostics, treatment and understanding causality.

### Limitations

We would like to address one major limitation of the potential study. Due to technical issues, we were unable to recruit a comparably sized control group. We have employed appropriate statistical tests robust for small and also unequal sample sizes. However, given the considerable individual variability of MMN these results are in need of replication.
